# Decellularization of Human Pancreatic Fragments with Pronounced Signs of Structural Changes

**DOI:** 10.3390/ijms24010119

**Published:** 2022-12-21

**Authors:** Victor I. Sevastianov, Anna S. Ponomareva, Natalia V. Baranova, Lyudmila A. Kirsanova, Yulia B. Basok, Evgeniy A. Nemets, Dmitry N. Kruglov, Igor A. Miloserdov, Sergey V. Gautier

**Affiliations:** 1 The Shumakov National Medical Research Center of Transplantology and Artificial Organs, 123182 Moscow, Russia; 2 The Institute of Biomedical Research and Technology (IBRT), Autonomous Non-Profit Organization, 123557 Moscow, Russia; 3 Department of Transplantology and Artificial Organs, Faculty of Medicine, The Sechenov University, 119991 Moscow, Russia

**Keywords:** pancreas, extracellular matrix, decellularization, tissue-specific scaffold, human adipose derived stem cell, pancreatic islets

## Abstract

A significant lack of donor organs restricts the opportunity to obtain tissue-specific scaffolds for tissue-engineering technologies. One of the acceptable solutions is the development of decellularization protocols for a human donor pancreas unsuitable for transplantation. A protocol of obtaining a biocompatible tissue-specific scaffold from decellularized fragments with pronounced human pancreas lipomatosis signs with preserved basic fibrillary proteins of a pancreatic tissue extracellular matrix was developed. The scaffold supports the adhesion and proliferation of human adipose derived stem cell (hADSCs) and prolongs the viability and insulin-producing function of pancreatic islets. Experiments conducted allow for the reliance on the prospects of using the donor pancreas unsuitable for transplantation in the technologies of tissue engineering and regenerative medicine, including the development of a tissue equivalent of a pancreas.

## 1. Introduction

The development and creation of pancreatic tissue equivalents in order to replenish the pool of β-cells impacted following autoimmune damage associated with Type I diabetes is hindered by problems related to the maintenance of viability of functionally active isolated pancreatic islets. During isolation, the islets are affected by a number of damaging factors such as ischemia, oxidative stress, or a possible enzyme cytotoxic action [1,2]. Additionally, the loss of vascularization, innervation as well as of the native extracellular matrix (ECM) may negatively impact the viability of isolated islets [2,3]. The cell-secreted ECM is a network of macromolecules including polysaccharide glycosaminoglycans and proteins (collagens, laminins, fibronectin), and is an important component of the tissue microenvironment [4,5]. By participating in the processes of intracellular transfer of signals of differentiation, gene expression, adhesion, migration and proliferation, the ECM facilitates the islets’ structural integrity, which is a necessary condition of their viability and secretory function [5,6,7,8,9]. During the post-isolation period, it is important to create a microenvironment for the islets typical of a native ECM in situ. Scaffolds capable of imitating the structure and composition of a natural pancreatic ECM may facilitate the preservation of the islets’ structure and functions in vitro and in vivo.

When creating pancreatic tissue equivalents, it is preferable to use bioresorbable scaffolds on the basis of the so-called ECM synthetic and biological mimetics and their composites [10,11,12,13,14].

In order to create tissue-engineered constructs with islet cells, collagen-containing hydrogels are also used [5,15]. Among the biomimetics modeling the ECM composition is a biopolymer microheterogeneous collagen-containing a hydrogel scaffold (BMCH scaffold, Sphero^®^GEL trademark, AO BIOMIR, Moscow, Russia)-a multi-component product containing natural substances including peptides of partially hydrolyzed collagen, glycoproteins, uronic acids, as well as ECM biomolecules [16]. It was shown that during incubation with collagen-containing scaffolds, the islets retain integrity, viability and secretory function for a long time versus the islet monoculture [17,18,19].

Despite their benefits, resorbable scaffolds from biopolymer materials are not tissue-specific, lacking the typical properties of structure and composition. Lately, as a prospective approach, the development of tissue-engineered constructs began based on tissue-specific scaffolds made from decellularized tissue [16,19,20,21,22] with their subsequent recellularization [14,20,23,24,25].

The development of effective protocols of pancreatic decellularization is directed at the utmost possible retention of structural, biochemical and mechanical properties of a native ECM with the maximally complete removal of cell material, including DNA and native tissue cell surface antigens [4,20,26,27]. The presence of main ECM components in decellularized pancreatic scaffolds, such as structure proteins (Type I, III, IV, V and VI collagen, elastin, fibronectin and laminin), glycoproteins, and cell adhesion factors, allows for the creation of conditions for a prolonged functional activity of islet (insulin-producing) cells and the maximal imitation of ECM properties [23,25,28]. The most complete removal of cellular material from tissue during decellularization leads to a minimized immune response during the further implantation of tissue-engineered constructs [29].

Decellularization protocols should also take into account such characteristics of native tissue as its density and lipid content [25]. Identical tissues may have varying structure and composition properties depending on an individual donor. Therefore, the optimization of a decellularization protocol is of primary importance in each separate case.

Research exists on obtaining decellularized scaffolds from pancreatic tissue and their subsequent successful recellularization [8,23]. An increase in insulin secretion by porcine pancreatic islets [21] and rat islets [19] cultured in the presence of tissue-specific scaffolds versus islets cultured alone was reported. Mirmalek-Sani et al. successfully decellularized a porcine pancreas retaining all main structure proteins, including various types of collagen, elastin, fibronectin and laminin [21]. ECM native proteins prevent β-cell apoptosis [30], binding, preserving and regulating the activity of growth factors which play an important part in the islets’ viability and functional activity. It was shown that a three-dimensional structure of BCM native components determines the topographic location of pancreatic endocrine cells which impact the islets’ viability and secretory activity [31]. Thus, upon the recellularization of a decellularized pancreas with the islets, the increased secretion of insulin versus isolated islets was achieved. The diabetes experimental model demonstrated that pancreatic tissue recellularized with the population of MIN-6β-cells is capable of controlling blood glucose levels in mice [32].

Thus, tissue-specific scaffolds made from a decellularized pancreas or pancreatic tissue fragments [14,19,23,24] which retain the properties of ECM native tissue are appropriate to create a tissue equivalent of an endocrine part of a pancreas [8,25].

Xenogeneic and allogeneic scaffolds of various origins exist. However, animal xenogeneic tissue may possess residual immunogenicity and be contaminated with biological agents. Human allogeneic tissue is an ideal source from which to obtain tissue-specific scaffolds. Unfortunately, a significant lack of donor organs for transplantation restricts their use for tissue engineering technologies. One of the acceptable solutions is the development of decellularization protocols for a donor human pancreas which is not suitable for transplantation due to its condition.

The aim of the study is the development of a decellularization protocol for fragments of a human pancreas with manifested signs of structural changes with the subsequent study of biological and functional properties of the obtained tissue-specific scaffold.

## 2. Results

### 2.1. Morphological Analysis

#### 2.1.1. Morphological Analysis of the Pancreas

**Figure 1:**
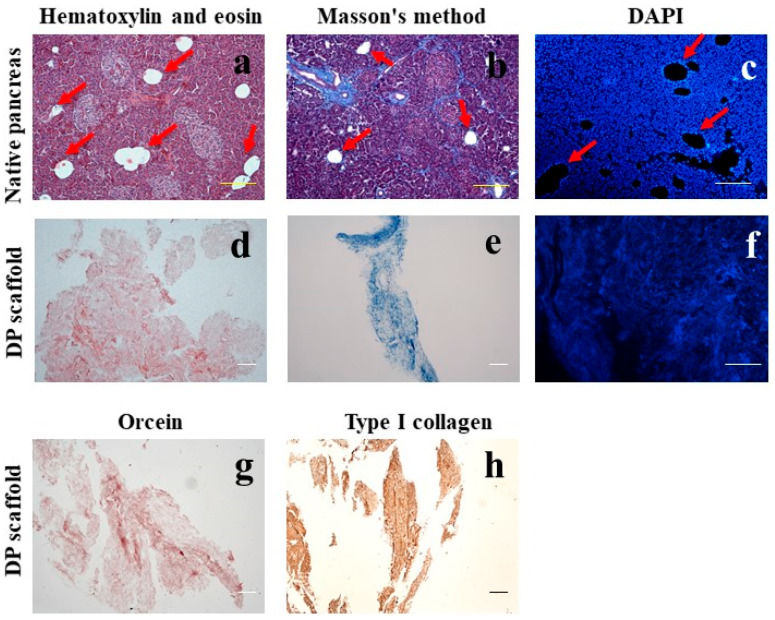
A histological picture of a native human pancreas with signs of lipomatosis (**a**–**c**) and of a tissue-specific scaffold from a decellularized pancreas with lipomatosis (DP scaffold) (**d**–**h**). Fat inclusions are marked with red arrows. Staining with hematoxylin and eosin (**a**,**d**), for total collagen by the Masson’s method (**b**,**e**), DAPI (**c**,**f**), for elastic fibers with orcein (**g**). Immunohistochemical staining with antibodies to type I collagen (**h**). Bar 100 µm.

As a result of a morphological analysis of a native pancreas, clear signs of lipomatosis were detected in pancreatic tissue (Figure 1a–c). In the pancreas samples, round or elongated islets were found with a compact or lobulated structure. Specific DAPI staining confirmed the presence of cellular nuclei both in the islets and in the surrounding acinar tissue.

#### 2.1.2. Morphological Analysis of the DP Scaffold

In the samples of pancreatic fragments with lipomatosis after three subsequent freeze-thaw cycles and detergent treatment, a complete absence of retained cells, separate cell nuclei as well as small fragments of cell detritus were observed in the obtained connective-tissue scaffold (Figure 1f). The obtained tissue was characterized by a porous, small-meshed, fine-grained structure (Figure 1d) with the preservation of collagen fibers (Figure 1e). The immunohistochemical staining also confirmed the presence of type I collagen in the DP scaffold, which is an important component of the pancreatic ECM (Figure 1h). With orcein staining, red-brown elastic fibers were noted. (Figure 1g). These results point to the effectiveness of this protocol of decellularizing a pancreas with the signs of lipomatosis and obtaining a well-purified scaffold with the preserved basic fibrillary proteins of the pancreatic tissue ECM.

### 2.2. DNA Content in the DP Scaffold

**Figure 2:**
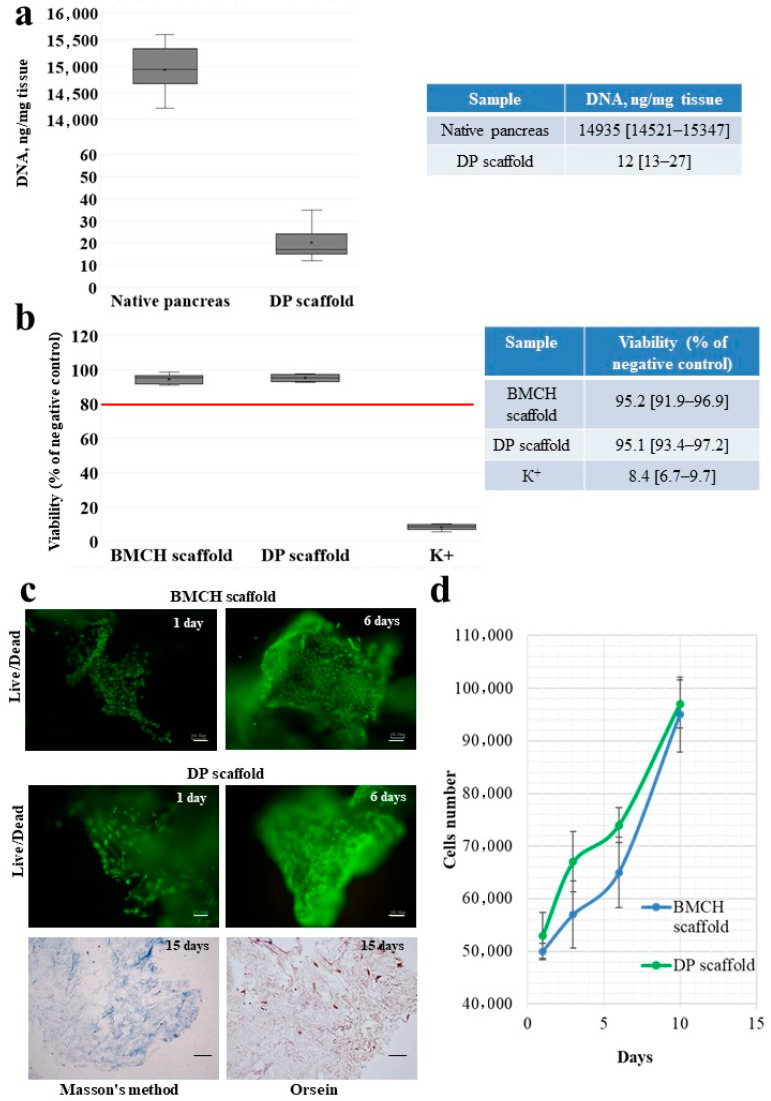
The amount of DNA of native pancreatic tissue and decellularized scaffold obtained from a pancreas with lipomatosis (**a**); *p* < 0.05. The metabolic activity of the NIH 3T3 fibroblasts (cytotoxicity test) after contact with a biopolymer microheterogeneous collagen-containing hydrogel scaffold (BMCH scaffold) and a tissue-specific scaffold (DP scaffold); K^+^–single element aqueous zinc standard 10,000 μg/mL; *p* < 0.05 (**b**). The adhesion and proliferation of hADMSC on BMCH and DP scaffolds. Masson’s, LIVE/DEAD^®^ and orcein staining (**c**). hADMSC growth curves on BMCH and DP scaffolds (**d**). Bar 100 µm.

The DNA content in the samples of native pancreatic tissue and the decellularized scaffold is presented in Figure 2a.

In the pancreatic tissue samples, DNA amounted to 14,935 [14,521–15,347] ng/mg of tissue. In the samples of the tissue-specific scaffold, no more than 0.1% of DNA is retained and less than 50.0 ng/mg of tissue is retained, which indicates the high effectiveness of the developed decellularization protocol of a pancreas with lipomatosis and the low immunogenicity of the obtained scaffold.

### 2.3. Cytotoxicity of the DP Scaffold

The fibroblast metabolic activity after direct contact with the DP scaffold in relation to the culture medium is presented in Figure 2b.

A cytotoxicity testing of a DP scaffold demonstrated that a tissue-specific scaffold does not have a cytotoxic impact on a cell culture.

### 2.4. Adhesion and Proliferation of MSC on the Scaffolds

The properties of the obtained tissue-specific scaffold to maintain cell adhesion and proliferation were studied on an hADSC culture. In order to compare the cell growth dynamics, a BMCH scaffold permitted for clinical practice was used.

On day 1 of culturing, hADSC adhesion and the spread of cells on the surface of a DP scaffold was observed much like with a BMCH scaffold (Figure 2c).

By day 6, the number of hADSCs has significantly increased, and cell growth areas were observed practically along the entire scaffold surface, which indicated their high proliferative activity (Figure 2d).

A histological analysis revealed that after 15 days of culturing, an intensive colonization of a decellularized hADSC scaffolds occurred. Flattened cells of a typical fibroblast-like shape were located not only on the scaffold’s surface but also actively penetrated its deep layers (Figure 2d).

### 2.5. Freshly Isolated Islets

**Figure 3:**
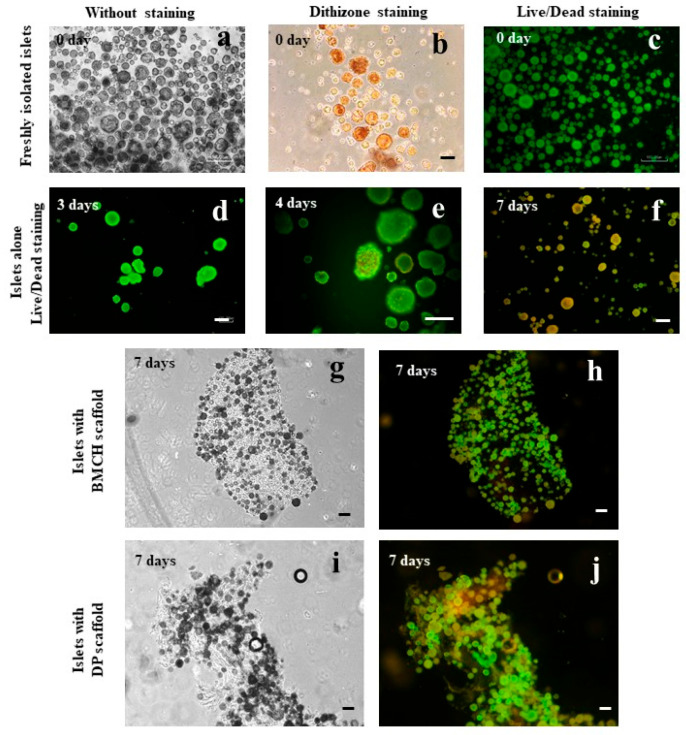
Freshly isolated human islets (**a**–**c**); islets cultured without scaffolds for 3, 4, 7 days–control group inverted microscopy (**d**–**f**); islets cultured with BMCH scaffold on day 7 experimental group I (**g**,**h**); islets cultured with DP scaffold on day 7 experimental group II (**i**,**j**). Phase contrast microscopy (**a**,**g**,**h**); dithizone staining (**b**); fluorescencence LIVE/DEAD^®^ (**c**–**f**,**h**,**j**). Bar 100 μm.

Following isolation, a significant amount of islets of various size were observed with a primarily round shape and a smooth surface (Figure 3a). Islets were identified by dithizone staining. The stain was selectively coloring these islets red and orange, while acinar cells remained unstained (Figure 3b). Live staining of LIVE/DEAD^®^ freshly isolated islets demonstrated the viability of their majority (Figure 3c).

### 2.6. Viability of Cultured Islets

#### 2.6.1. Islets Alone (Control Group)

The majority of islets produced without scaffolds (control group) retained their shape and integrity during the first three days of incubation. Some islets had signs of fragmentation or were destroyed.

The LIVE/DEAD^®^ staining displayed green fluorescence, confirming the islets’ viability (Figure 3d). After 3 days of culturing, the morphology in the control group changed. In some islets, cavities and signs of fragmentation were detected, and the surface of a significant number of islets became uneven (Figure 3e). In the remaining islets, the LIVE/DEAD^®^ staining at 4–7 days of culturing detected dead cells with red fluorescence (Figure 3f). Thus, after a week of culturing without scaffolds, the islets underwent significant destructive changes.

#### 2.6.2. Islets with Biopolymer Scaffold

The islets cultured with a BMCH scaffold (experimental group I) remained intact without fragmentation or degradation during the entire observation period (7 days). On day 2 of incubation an islet adhesion to the smooth surface of the BMCH scaffold was observed (Figure 3g), while some islets free-floated in the culture medium. The fluorescent LIVE/DEAD^®^ staining performed on day 1, 4 and 7 of incubation confirmed the viability of the experimental group I islets (Figure 3h).

#### 2.6.3. Islets with Tissue-Specific Scaffold

The islets cultured with a DP scaffold (experimental group II), much like those in the experimental group I, did not display any signs of destruction or fragmentation during the entire observation period (7 days). On day 2 of incubation with a DP scaffold, the majority of islets displayed adhesive properties and deposited on the scaffolds’ fibrous surface (Figure 3i) while the islets left in the culture medium continued to float. The LIVE/DEAD^®^ staining of the experimental group II islets conducted on day 1, 4 and 7 of incubation confirmed the viability of the remaining islets (Figure 3j).

### 2.7. Islet Insulin-Producing Function

**Figure 4:**
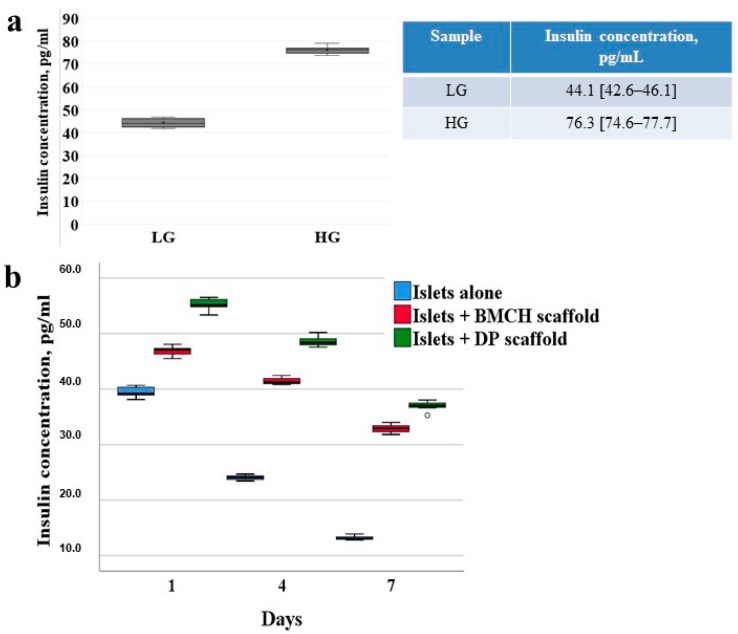
The functional activity of islets alone 1 day after isolation (**a**). The insulin-producing function of human islets in the control and experimental groups (**b**).

The value of insulin concentration after stimulation with glucose for a period of 24 h of culture islets alone increased by 1.7-fold (Figure 4a).

A comparative analysis of insulin secretion of the experimental groups I and II was conducted with the reference to the control group (Figure 4b). After the first 24 h of culturing, the concentration of insulin in experimental groups I and II was 20.2 % (47.11 [46.02–47.69] pg/mL) and 40.7 % (55.11 [54.25–56.29] pg/mL) higher versus the control (39.18 [38.61–40.40] pg/mL); on day 4 of incubation–71.2 % (41.23 [40.87–42.08] pg/mL) and 100.8 % (48.36 [47.75–49.45] pg/mL) higher, respectively, versus the control (24.09 [23.62–24.50] pg/mL). On the 7th day, an even greater increase in insulin concentration was observed in experimental group I by 152.0% (32.91 [32.14–33.62] pg/mL) and in experimental group II by 183.5% (37. 03 [36.14–37.79] pg/mL) compared with the control group (13.06 [12.85–13.56] pg/mL).

A positive influence of the scaffolds on the islets’ insulin-producing function was also displayed in the difference in the hormone’s concentration in the control and experimental groups during the entire study. The significant difference between the insulin concentrations of the experimental groups I and II and the control can be explained by destructive changes in the islets’ monoculture after three days of culturing, which is confirmed by the data of fluorescent staining at various time points. The percentage-wise positive trend of the impact by biopolymer and tissue-specific scaffolds on the islets’ secretory function was retained during the entire observation term despite the fact that the insulin concentration reflected in absolute values has been decreasing with the duration of culturing.

The level of insulin secretion on day 1 and 4 of islet culturing was higher in group II by 17.37% versus group I; and on day 7 it was higher by 11.43%. Thus, an insignificant advantage of using a tissue-specific DP scaffold versus a BMCH scaffold while culturing human pancreatic islets was revealed.

## 3. Discussion

Using a combination of physical and chemical decellularization methods, a tissue-specific scaffold from a human pancreas with signs of lipomatosis was obtained.

The protocols for the decellularization of a human pancreas have been described [22,25,33]; however, the decellularization of pancreatic tissue with various structural changes has not been previously described. The study of native tissue’s structure and composition such as density, rigidity, and elasticity, is an important aspect of optimizing the decellularization protocol [4]. For example, the inclusion of the delipidization stage has increased the gel-forming ability of the obtained scaffold [25]. It is possible that the physico-mechanical properties will correlate to a certain degree to histological properties of a pancreas. This suggestion requires further study.

In studies [21] and [22], decellularization of the whole pancreas was conducted using perfusion with Triton X-100 and NH_4_OH solution. In this study, we focused on the possibility of obtaining a finely dispersed scaffold from the fragments of pancreatic tissue obtained as a result of a pancreatic surgery. The obtained cytocompatible DP scaffold is suitable for use in vivo with insulin-producing cells in injection form. It is known that the decellularization is a rather complex and lengthy process [23,29]. There is a risk of an incomplete decellularization of a whole organ due to disruptions in a detergent’s microcirculation and the low recellularization of the entire scaffold’s volume and complications with oxygen and nutrients delivery to cells [34]. Using fragments of pancreatic tissue in order to obtain a scaffold allows for a decrease in these risks.

In one study [24], a porcine pancreas was minced and treated with a 1% solution of Triton X-100 with 0.1% NH4OH, followed by DNase treatment to obtain a decellularized scaffold with the remaining DNA of no more than 50 ng/mg of tissue. The DP scaffold obtained in our study contained no more than 0.1% DNA and corresponded to the recommended standard [29] without using DNase, which helped reduce the number of tissue treatment stages.

The decellularization methods included treatment with Triton X-100 and NH4OH solution [21,22,24] or treatment with deoxycholate [25]. Our protocol used Triton X100, which target lipid-lipid and lipid-protein interactions [24], and sodium dodecyl sulfate (SDS) to efficiently remove nuclear and cytoplasmic fragments [4].

The obtained scaffold in vitro facilitates both the islets’ viability and the maintenance of their secretory function for 7 days at a higher level versus the islets alone. Earlier, we researched the insulin-producing function of rat islets cultured in the presence of a BMCH scaffold and a scaffold from a rat’s decellularized pancreas [19]. A comparative analysis of insulin secretion revealed a more pronounced impact of researched scaffolds on rat islets versus human islets. That said, the rat islets’ insulin secretion level cultured in the presence of a tissue-specific scaffold was 35.5% higher versus the rat islets cultured in the presence of a BMCH scaffold.

## 4. Materials and Methods

### 4.1. Pancreas Retrieval and Preparation

The decellularized scaffold and the islets of Langerhans were obtained from the fragments of pancreatic tissue with prominent lipomatosis (n = 5). All manipulations were performed according to the WMA Declaration of Helsinki and approved by the Local Ethics Committee at the Shumakov National Medical Research Center of Transplantology and Artificial Organs, Moscow, Russia (16 March 2018, Protocol No. 160318-1/1e).

The procedure of islet isolation was performed no later than 10 h following the pancreas resection. The fragments of pancreatic tissue were frozen and stored at −80 °C for the subsequent decellularization.

### 4.2. Pancreas Decellularization

**Figure 5:**
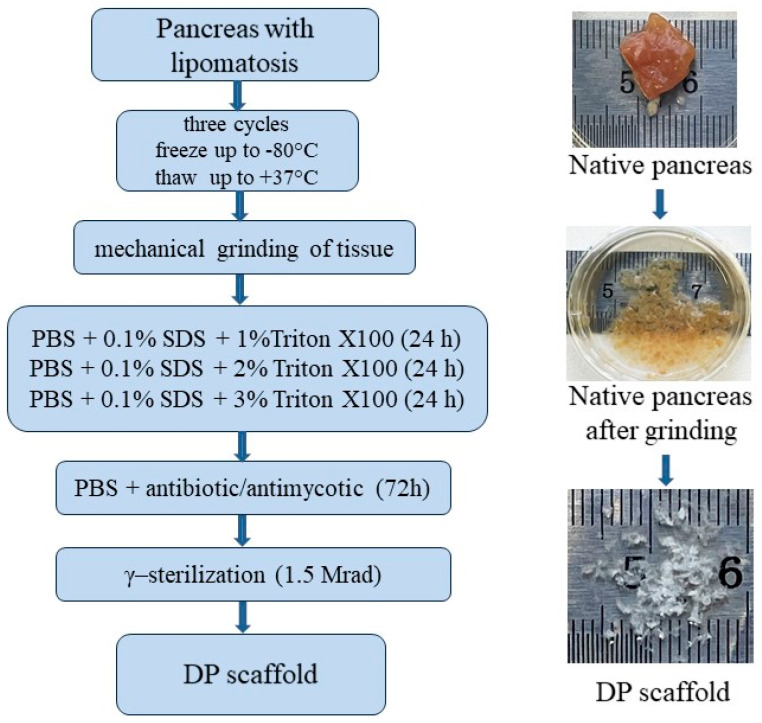
A flowchart for obtaining a tissue-specific scaffold (DP scaffold) from pancreatic tissue with lipomatosis.

Following the known decellularization methods of various parenchymal organs [4,8,23,25,29,33], a combination of physico-chemical methods for the decellularization of pancreatic tissue was found. The flowchart of pancreatic tissue decellularization is shown in Figure 5.

Pancreatic fragments with lipomatosis underwent three cycles of freezing up to −80 °C and thawing to +37 °C for the physical destruction of cell membranes and cell lysis (physical cell damage by freezing and thawing), with a subsequent mechanical tissue disintegration to 2 × 1 × 1 mm.

To dissolve cell membranes and dissociate detritus, surfactants were used: Triton X100 (Sigma-Aldrich, Inc., St. Louis, MO, USA), which targets lipid-lipid and lipid-protein interactions [24], and sodium dodecyl sulfate (SDS) to efficiently remove nuclear and cytoplasmic fragments [4]. Tissue fragments were treated in three changes of phosphate buffered saline (PBS) (pH = 7.4), containing 0.1% SDS solutions and increasing concentrations of Triton X100 (1%, 2% and 3%, respectively). In each solution, the sample was kept for a day at room temperature under the conditions of constant stirring at a speed of 1.0 rpm on the CellRoll roller system (INTEGRA Biosciences AG, Wallisellen Switzerland) to remove cell detritus [25,35]. The removal of remaining DNA is of primary importance during decellularization due to the nuclear material’s tendency to adhere to ECM proteins. During solution change, tissue fragments were filtered through a strainer with a 100-µm cell diameter and returned to a solution with a higher Triton X100 concentration.

Following the decellularization process, tissue was thoroughly rinsed from the remnants of surfactant agents for 72 h in three changes of antibiotic-antimicotic containing PBS (ampicillin, 10.0 μg/mL, and amphotericin B, 1.5 μg/mL). The obtained samples of the finely dispersed scaffold (DP scaffold) were placed in 10-mg cryotubes, frozen and γ-sterilized (1.5 Mrad).

### 4.3. Histology Staining

Fresh (n = 25) and decellularized (n = 15) tissue samples, previously fixed in 10% buffered formalin, were dehydrated in alcohols of increasing concentration, kept in a solution of chloroform and ethanol/chloroform, and finally embedded in paraffin. The samples were sliced into 4–5 µM sections using a Leica RM2245 microtome (Leica biosystems). Sections were stained at room temperature by Haematoxylin and Eosin (H&E), by Masson’s method, by orsein staining according to the Unna-Tentzer method, and DAPI fluorescent staining (Sigma-Aldrich, Inc., MO, USA).

Additionally, an immunohistochemical reaction was performed on Type I collagen with the anti-collagen I antibody (Abcam, Cambridge, UK) and the Rabbit Specific HRP/DAB (ABC) Detection IHC kit imaging system (Abcam, UK).

### 4.4. DNA Quantification

The residual amount of DNA was determined in the native tissue and DP scaffold samples. Freeze-dried native tissue (n = 7) and the DP scaffold (n = 7) samples weighing 10 mg were digested with Lysis Buffer (Buffer AL, catalog number 19075, QIAGEN, Hilden, Germany) and proteinase K for 16 h at +57 °C. The DNA was then extracted using the QIAGEN DNAeasy Blood and Tissue Kit according to the manufacturer’s instructions. The quantitative DNA determination with ™Picogreen Quant-iT (Thermo Fisher Scientific, Waltham, MA, USA) flourescent dye was used according to the protocol. To sum up, 50 μL of a sample’s lysate was dissolved in the 1:1 proportion with the TE buffer solution and then added to 100 μL of a staining solution. For a period of 5 min, the obtained solution was incubated at room temperature with no exposure to light, then activated with 480-nm emittance and analyzed with a Spark 10M microplate reader (Tecan Trading AG, Männedof, Switzerland) at a wavelength of 520 nm. In order to determine the absolute quantity of DNA, the DNA bacteriophage λ analytical curve was used (Thermo Fisher Scientific, MA, USA) (0 ng/mL–1000 ng/mL).

### 4.5. Biopolymer Microheterogeneous Collagen-Containing Hydrogel (The BMCH Scaffold)

As a control scaffold in experiments with cells and islets, an injectable form of a biopolymer microheterogeneous collagen-containing hydrogel (BMCH scaffold) was used. The injectable form of the BMCH scaffold (trademark Sphero^®^GEL, JSC Biomir Service, Moscow, Russia) registered in Russia for clinical use is produced from tissue components of farm animals using the method of acetic acid extraction. The BMCH scaffold contains the main ECM components: peptides of partially hydrolyzed collagen, glycoproteins and uronic acids, as well as growth factors necessary for vital cell activity, and the synthesis of exogenous uronic acids, proteoglycans and collagen [16,19].

### 4.6. Cytotoxicity

The cytotoxicity of the DP scaffold samples (n = 7) in vitro was evaluated by direct contact according to the ISO 10993-5:1999 international standard on the mouse embryonic fibroblast line NIH/3T3 [36]. The 10% fetal calf serum culture medium (HyClone, SV30160.03, Cytiva, Marlborough, MA, USA) served as the negative control. The positive control sample was a single-element aqueous standard of 10,000 μg/mL zinc (Sigma-Aldrich, USA). Culture monitoring was performed with the Eclipse TS100 (Nikon, Tokyo, Japan) inverted microscope. The metabolic activity of fibroblasts after contact with scaffold samples was determined after 24 h with the prestoBlue™ Cell Viability Reagent (Thermo Fisher Scientific, MA, USA) according to the protocol recommended by the manufacturer.

The proliferating cells rebuild prestoBlue™, resulting in a color change from indigo to pink. The percentage of recovered prestoBlue™ characterizes the metabolic activity of the cells. The changes in media absorption were recorded using a Spark 10M microplate reader (TecanTrading AG, Männedof, Switzerland) with Spark Control™ Magellan V1.2.20 software at a wavelength of 570 nm and 600 nm. The data quantitative and statistical processing was performed with an SPSS26.0. All results are presented as a mean ± standard deviation. The differences were considered significant at *p* < 0.05.

### 4.7. Cell Isolation, Adhesion and Proliferation

The ability of a DP scaffold to support cell adhesion and proliferation was studied on the human adipose derived stem cell (hADSC) culture.

The hADSCs were isolated from healthy donors’ adipose tissue during liver transplantation in accordance with the protocol approved by the Local Ethics Committee at the Shumakov National Medical Research Center of Transplantology and Artificial Organs, Moscow, Russia (the 24 January 2020 Protocol No. 240120-1/1e). Adipose tissue samples were washed extensively with PBS, minced and treated with collagenase type IA (600 units/g) (Sigma, USA) for 30 min at 37 °C. The enzyme was inactivated with an equal volume of MesenPRO^®^ medium (Thermo Fisher Scientific, USA) and centrifuged for 15 min at 400× *g*. The cellular pellet was resuspended and cultured under humid conditions, at 37 °C, and in a 5% CO_2_ atmosphere. The hADSCs of two passages were used for experiments.

The cells were cultured on tissue-specific (n = 5) and biopolymer (n = 5) scaffolds at 5 × 10^4^ cells/10 mg of the scaffold. The evaluation of cells’ proliferative activity and the viability on the scaffolds was conducted on day 1, 3, 6 and 10 of the culture using vital stains: prestoBlue™ Cell Viability Reagent (Thermo Fisher Scientific, MA, USA) to build a growth curve; and a LIVE/DEAD^®^ Cell Viability/Cytotoxicity Kit (Thermo Fisher Scientific, MA, USA) to determine viability. After 15 days of culturing, scaffold samples with cells were fixated in 10% buffered formalin for the further histological analysis.

### 4.8. Islets Isolation and Dithizone Staining

In order to study the functional properties of a tissue-specific scaffold in vitro, human islets of Langerhans were isolated from surgical specimens using a collagenase method. Pancreatic tissue was minced and incubated in the NB1 collagenase solution (activity 20 PZ U/g of tissue) with neutral protease NP (activity 1.5 DMC U/g of tissue) (Serva, Heidelberg, Germany) during 10–15 min at 37 °C. The action of enzymes was halted by adding a three-fold volume of cold (4 °C) Hanks’ solution (Paneco, Moscow, Russia) with subsequent filtering through a metal strainer with a 0.4–0.6 mm cell diameter. In order to rinse the islets, a 1-min centrifuging regime was selected at a speed of 900 rpm with a subsequent 2 min at a speed of 1300 rpm.

The islets were identified using dithizone staining (Sigma-Aldrich, Inc., MO, USA) immediately following their isolation. To this end, part of the suspension was mixed with dithizone solution in the proportion of 2:1 and incubated for 20–30 min at 37 °C. Freshly isolated islets were resuspended in a growth medium and used for testing no later than 24 h after their isolation.

### 4.9. Islet Culture

An equal amount of isolated islets (~200) was placed in three 25 cm^2^ culture flasks (Greiner bio-one, Frickenhausen, Germany). The scaffold was not added to the first culture flask (control, n = 5). The second and third culture flasks included 20.0 ± 0.1 mg of a BMCH scaffold (experimental group I, n = 5) and 20.0 ± 0.1 mg of a DP scaffold (experimental group II, n = 5), respectively. All islets were cultured in a full growth medium containing DMEM (glucose 1.0 g/L) (Paneco, Russia), 10% fetal calf serum (HyClone, Cytiva, MA, USA), Hepes (Thermo Fisher Scientific, USA), 2 mM L-glutamine (Paneco, Moscow, Russia), 1% antibiotic/antimycotic (Thermo Fisher Scientific, MA, USA).

Islets were cultured under standard conditions at 37 °C in a humid atmosphere containing 5% CO_2_ with daily visual monitoring and photography using an inverted microscope (Nikon, Tokyo, Japan) with a digital camera.

### 4.10. Cell Viability Assay

The viability of freshly isolated islets as well as of the islets cultured in control and experimental groups was evaluated on day 1, 4 and 7 using the LIVE/DEAD^®^ Cell Viability/Cytotoxicity Kit (Thermo Fisher Scientific, MA, USA). Islets alone or with scaffolds were placed in a Petri dish, mixed with the prepared staining solution at a 2:1 proportion and dark-incubated for 15–30 min. The result was evaluated using a luminescent microscope (Nikon TS-100, Tokyo, Japan).

### 4.11. Enzyme Immunoassay (ELISA)

Functional activity of the islets alone was determined under the influence of the traditional stimulator of insulin secretion—glucose. After one day of incubation, the growth medium was replaced with a fresh portion of the low glucose medium (2.8 mmol/L). The culture medium samples were taken after a 60-min incubation under the same conditions and frozen (−23 °C). The growth medium was then removed and replaced with a fresh high glucose medium (25 mmol/L). The samples of the growth medium after 60 min of incubation under the indicated conditions were also taken and frozen (−23 °C) for ELISA.

The basal insulin concentration was determined on the 1st, 4th and 7th days of culture in the control and experimental groups. The culture medium was replaced with a fresh one; sampling from all flasks was performed after 1 h of incubation under standard conditions (37 °C, 5% CO_2_). The samples were preserved in a frozen condition (−23 °C) for the subsequent enzyme immunoassay.

The concentration of insulin in the culture medium samples (n = 77) was determined by competitive inhibition using the ELISA Kit for insulin Human CEA448 Hu-96 (Cloud-Clone Corp., Texas, USA).

The results of the ELISA quantitative method were calculated measuring the optical density using the Spark 10M microplate reader (TecanTrading AG, Männedof, Switzerland) with the Spark Control™ Magellan V1.2.20 software at 450 nm and 550 nm wavelengths to account for the reader’s optical defects.

### 4.12. Statistical Analysis

Data were analyzed with SPSS26.0 statistical software. The distribution of variables was tested with the Shapiro–Wilk procedure. The results were compared using the Mann–Whitney unpaired *t*-test and Kruskal—Wallis test, where *p* < 0.05 was considered statistically significant.

## 5. Conclusions

A protocol of decellularizing the fragments of a human pancreas with manifested lipomatosis was developed. It was demonstrated that the obtained biocompatible tissue-specific scaffold retains the basic properties of the ECM native tissue and is capable of prolonging the insulin-producing function of human islets in vitro. The results of the current study allow us to hope for prospects in using a human donor pancreas unsuitable for transplantation in the technologies of tissue engineering and regenerative medicine, including in the development of a pancreatic tissue equivalent.

## Data Availability

Not applicable.
